# Clinical impact and safety of brain biopsy in unexplained central nervous system disorders: a real‐world cohort study

**DOI:** 10.1002/acn3.70000

**Published:** 2025-02-21

**Authors:** Robin W. van Steenhoven, Saan Salih, Juna M. de Vries, Ide Smets, Rob M. Verdijk, Mayke Gardeniers, Jeroen Kerstens, Juliette Brenner, Yvette S. Crijnen, Marjolein Geurts, Jacoline E. C. Bromberg, Corine H. GeurtsvanKessel, Peter A. E. Sillevis Smitt, Rutger K. Balvers, Maarten J. Titulaer

**Affiliations:** ^1^ Department of Neurology Erasmus University Medical Center Rotterdam The Netherlands; ^2^ Department of Neurosurgery Erasmus University Medical Center Rotterdam The Netherlands; ^3^ Department of Neuropathology Erasmus University Medical Center Rotterdam The Netherlands; ^4^ Department of Radiology Erasmus University Medical Center Rotterdam The Netherlands; ^5^ Department of Viroscience Erasmus University Medical Center Rotterdam The Netherlands

## Abstract

**Objective:**

A substantial part of central nervous system (CNS) disorders remains unexplained, despite various new and minimally invasive diagnostic techniques. Within this rapidly developing diagnostic field, the precise role of brain biopsy is unknown. We aimed to study the clinical impact and safety of brain biopsies in unexplained CNS disorders.

**Methods:**

In this retrospective cohort study, we included all adult patients who were referred for a diagnostic work‐up to our academic center with neuro‐inflammatory, neuro‐oncological, and neuro‐infectious expertise and underwent a brain biopsy between January 2010 and December 2023. Typical cases of CNS neoplasms and infections were not analyzed. Brain biopsies were evaluated with respect to diagnostic and therapeutic impact and complication risk.

**Results:**

Brain biopsy was performed in 587 patients. Ninety‐four patients with a CNS disorder of unknown cause, with 107 biopsies, were analyzed (44% female, median age 58 years). Postoperative diagnoses included brain tumors/lymphomas (37/94, 39%), inflammatory disorders (11/94, 12%), infections (8/94, 9%), autoimmune encephalitis (8/94, 9%), and primary angiitis of the CNS (4/94, 4%). Diagnostic yield of brain biopsy was 62%, increasing up to 72% after repeat biopsies, as 10 additional patients were diagnosed with a brain tumor. In 77% of patients, brain biopsy changed the treatment strategy. Symptomatic intracranial hemorrhage occurred in 4 of 107 brain biopsies (4%).

**Interpretation:**

In a selected population of patients with unexplained CNS disorders, clinical impact of brain biopsies is high, while being relatively safe. A multidisciplinary team approach is fundamental in establishing optimal indication for brain biopsy and subsequent treatment decisions.

## Introduction

The diagnostic work‐up of rare and atypically presenting central nervous system (CNS) disorders is often complex and time‐consuming, as the differential diagnosis includes both inflammatory, infectious and oncological disorders, requiring multiple investigations. The diagnostic landscape of CNS disorders has significantly improved in the past decades, as various new and relatively minimally invasive diagnostic tests were introduced, including neuronal autoantibody testing in autoimmune encephalitis (AE), real‐time quaking‐induced conversion (RT‐QuIC) in Creutzfeldt–Jakob disease (CJD), intracranial vessel wall imaging in vasculopathies and metagenomic next generation sequencing (mNGS) in infections.^1^, ^2^, ^3^, ^4^ Unfortunately, a substantial part of CNS disorders still remains unexplained.^5^ In these patients, clinicians are split between initiation of empirical treatment and referral for a brain biopsy, in particular if rapid neurological deterioration is present. The clinical utility of brain biopsy has been well‐established in brain tumors (diagnostic yield ~95%) and CNS lesions in immunocompromised patients.^6^, ^7^, ^8^, ^9^ However, in unexplained CNS disorders, the precise role of brain biopsy is unknown and clinical guidelines are not available.

Generally, brain biopsy is considered a last resort given its invasive character and uncertain diagnostic yield.^10^ It is debatable whether this consideration is appropriate for multiple reasons. First, delay of a correct diagnosis and targeted treatment due to multiple investigations prior to brain biopsy may result in more permanent brain injury and a worse outcome, especially in rapidly progressing syndromes.^11^, ^12^ Second, histological confirmation is mandatory for the diagnosis of some, relatively new diseases. For instance, brain biopsy can be used to fulfill the ancillary testing feature, in addition to MRI and CSF testing, of the 2016 clinical criteria for seronegative AE.^3^ Third, in brain tumors, relatively few symptomatic complications from biopsies are observed, suggesting that the risk of brain biopsy in unexplained CNS disorders might be overestimated.^13^ Conversely, it should be taken into account that the histological findings in non‐neoplastic disorders might be less specific compared to brain tumors and their interpretation requires a multidisciplinary approach.^12^, ^14^ In particular the establishment of neuroinflammatory diagnoses using brain biopsy is challenging, as some nonspecific inflammatory abnormalities are also observed in various other diseases (e.g., neoplasms).^15^

Various earlier studies reported the diagnostic yield of brain biopsy in unexplained CNS disorders.^16^, ^17^ Unfortunately, descriptions on indication, histological interpretation and clinical implications are often limited, although these elements are vital for a successful brain biopsy. In this study, we aimed to comprehensively describe the clinical impact and safety of brain biopsies in unexplained CNS disorders.

## Subjects/Materials and Methods

### Patients

In this retrospective cohort study, we included all adult patients (≥18 years) who underwent a brain biopsy between January 2010 and December 2023 at the Erasmus University Medical Center, a national center of expertise for neuroinflammatory disorders and neuro‐oncology (defined as total cohort). Patients with a space occupying lesion, showing typical features of a tumor or abscess, specifically referred for a brain biopsy to establish treatment strategy (e.g., confirmation of tumor type or microorganism), were not analyzed (defined as typical cohort). Remaining patients with unknown preoperative diagnoses were defined as the atypical cohort and analyzed. Brain tumor patients from the atypical cohort were compared to brain tumor patients from the typical cohort (ratio 1:3), matched for tumor type, sex, and age (±5 years). This study was performed according to the Strengthening the Reporting of Observational Studies in Epidemiology (STROBE) reporting guideline for observational research.^18^ IRB approval was waived, but informed consent was obtained from patients or their caregivers, if possible.

### Diagnostic work‐up and operative technique

All patients were clinically evaluated in person by the authors. The diagnostic work‐up was individualized per patient, but generally included blood analysis, brain MRI, EEG, lumbar puncture and, if indicated, total‐body CT or FDG‐PET/CT with subsequent biopsy of systemic lesions, when present. All patients were preoperatively discussed in a multidisciplinary panel, including neuro‐oncologists, neuroimmunologists, neurosurgeons, and neuroradiologists to establish the indication of brain biopsy. All brain biopsies were lesional, defined as the presence of a clear anatomical abnormality on brain MR, which was identified by a qualified neuroradiologist using standardized MRI sequences, minimally including T1, T2, T2‐weighted fluid‐attenuated inversion recovery (FLAIR), T1 with contrast and diffusion weighted imaging (DWI). In general, stereotactic biopsy was used for deep‐seated lesions and open biopsy for superficial cortical lesions. Medtronic Stealth TreonTM Vertek^®^ or BrainLAB^®^ Varioguide frameless stereotactic system was used, as described previously.^9^ Four biopsies were obtained at the biopsy target, in addition to two to four biopsies proximal to the biopsy target. In open‐brain biopsy, ~1 cm^3^ specimen of arachnoid, pia, cortex, and underlying white matter was obtained through a burr hole or craniotomy. Tissue samples were sent for neuropathological examination and, if indicated, additional molecular, immunological and microbiological testing. A NGS panel targeting mutated genes diagnostic of glioma was implemented as part of routine diagnostics in suspected glioma in our institute in 2013, assessing mutations in ATRX, CIC, EGFR, FUBP1, NOTCH1, PTEN; H3F3A, IDH1/2, PIK3CA, TERT, and BRAF, amplifications in EGFR or MDM2, and copy number alterations of chromosome 1p, 7, 10, and 19q.^19^ Postoperatively, CT brain was performed in case of neurological deterioration. Neuropathological results and clinical implications were discussed multidisciplinary.

### Diagnostic yield and clinical impact

Medical files and pathological reports were reviewed by two neurologists (RVS, MT), a neurosurgeon (RB), and a research intern (SS). Postoperative diagnoses were established by integration of preoperative diagnostic work‐up and histological diagnoses, whereas follow‐up and autopsy, if applicable, were also reviewed for final diagnoses. Established criteria and classifications were applied to define diagnostic categories.^3^, ^20^, ^21^, ^22^, ^23^ Brain biopsy was considered diagnostic if a significant contribution to the final diagnosis was provided, either by obtaining a specific histological diagnosis or less specific findings in combination with exclusion of alternative diagnoses. Accordingly, therapeutic impact was defined as a significant influence on treatment decisions, also if brain biopsy allowed safe initiation of immunotherapy by excluding other diagnoses. In case of repeat brain biopsies, the final biopsy was used to determine diagnostic yield and therapeutic impact. Complications of all brain biopsies within <30 days postoperative were evaluated and classified according to an acknowledged grading scale for neurosurgical complications.^13^

### Statistics

We used IBM SPSS 25.0 (SPSS Inc) and R statistical software (v4.3.2; R Core Team 2023) for statistical analysis.^24^ Pearson chi‐squared test or the Fisher–Freeman–Halton test, when appropriate, were used for patient characteristics analysis and group comparisons, encompassing categorical data. *P*‐values were two‐sided and considered statistically significant when below 0.05. No correction was applied for multiple testing so all *P*‐values between 0.05 and 0.005 should be considered with caution.

## Results

In total, brain biopsy was performed in 587 patients between January 2010 and December 2023 (total cohort), of whom 94 (16%) had a CNS disorder of unknown cause and were analyzed (atypical cohort; Fig. S1). In the other 493 patients, the lesions were clearly space occupying and of neoplastic or infectious nature (typical cohort; 489/587; 83.5%) or insufficient information was available (4/587; 0.5%). In the atypical cohort, 41 patients (44%) were female and median age was 58 years (interquartile range [IQR] 50–69, range 19–79; Table 1). Immunocompromised status was observed in 19 of 94 patients (20%). All patients had a radiologically identifiable lesion. In total, 107 brain biopsies were performed, as 13 repeat biopsies were performed in 11 of 94 patients (12%; Table 2). Median time between onset of symptoms and first brain biopsy was 23 weeks (IQR 6–30; 0–780). Fifty‐one out of 94 biopsies (54%) were stereotactic biopsies (Table 2).

**Table 1:** Clinical characteristics of patients with unexplained CNS disorder referred for brain biopsy (atypical cohort).

	*N* = 94
Female gender, *n* (%)	41 (44)
Age at biopsy in years, median; IQR; range	58; 50–69; 19–79
Past medical history, *n* (%)	
Autoimmune disease	28 (30)
Systemic malignancy	14 (15)
Immunocompromised status	19 (20)
Initial presentation, *n* (%)	
Subacute onset	87 (93)
Focal deficits	48 (51)
Working memory deficits	40 (43)
Behavioral symptoms	36 (38)
New‐onset seizures	33 (35)
Impaired consciousness	16 (17)
Cerebellar ataxia	14 (15)
Brainstem symptoms	12 (13)
MRI brain performed, *n* (%)	93^a^ (99)
Cortical	59 (63)
Mesiotemporal	23 (25)
Unilateral	14 (15)
Bilateral	9 (10)
Subcortical and white matter	53 (56)
Deep gray matter	28 (30)
Brainstem	22 (23)
Cerebellum	12 (13)
Meninges	18 (19)
Increased T2/FLAIR signal	85 (90)
Enhancement	73 (78)
Restricted diffusion	28/93^a^ (30)
EEG performed, *n* (%)	27 (29)
Epileptic abnormalities	2/27 (7)
CSF performed, *n* (%)	77 (82)
WBC >5/μL	38/77 (49)
CSF‐specific oligoclonal bands	13/54 (24)
Systemic features, *n* (%)	
Tumor	2 (2)
Lymphadenopathy	14 (15)
Preoperative corticosteroids, *n* ^b^ (%)	29 (31)
Interval last corticosteroid and biopsy in days (median; IQR; range)	13; 0–36; 0–59
Modified Rankin Scale, mRS; median; IQR; range	3; 2–4; 1–5

CNS, central nervous system; CSF, cerebrospinal fluid; FLAIR, fluid‐attenuated inversion recovery; IQR, interquartile range; WBC, white blood cell count.

^a^ CT with contrast was performed in one patient due to pacemaker not compatible with MRI.

^b^ <60 days prior to brain biopsy.

**Table 2:** Characteristics of performed brain biopsies in patient with unexplained CNS disorder.

Total no. of brain biopsies (in 94 patients)	107
Duration between onset and first biopsy in weeks, median; IQR; range	23; 6–30; 0–780
No. of patients per category (%)	
One biopsy	83/94 (88)
Two biopsies	9/94 (10)
Three biopsies	2/94 (2)

Biopsy technique, *n* (%)	Stereotactic	Open
All biopsies	57/107 (53)	50/107 (47)
First biopsy	51/94 (54)	43/94 (46)
Second biopsy	5/11 (45)	6/11 (55)
Third biopsy	1/2 (50)	1/2 (50)

Target of brain biopsy, *n* (%)	First biopsy (*n* = 94)	Second biopsy (*n* = 11)	Third biopsy (*n* = 2)
Cortical	47 (50)	3 (27)	0 (0)
Corticomeningeal	16/47 (34)	1/3 (33)	0 (0)
Subcortical	34 (36)	6 (55)	2 (100)
Deep gray matter	5 (5)	0 (0)	0 (0)
Brainstem/cerebellum	8 (9)	2 (18)	0 (0)
Enhancement	73 (78)	9 (82)	2 (100)

CNS, central nervous system; IQR, interquartile range.

### Diagnostic yield and therapeutic impact

**Figure 1:**
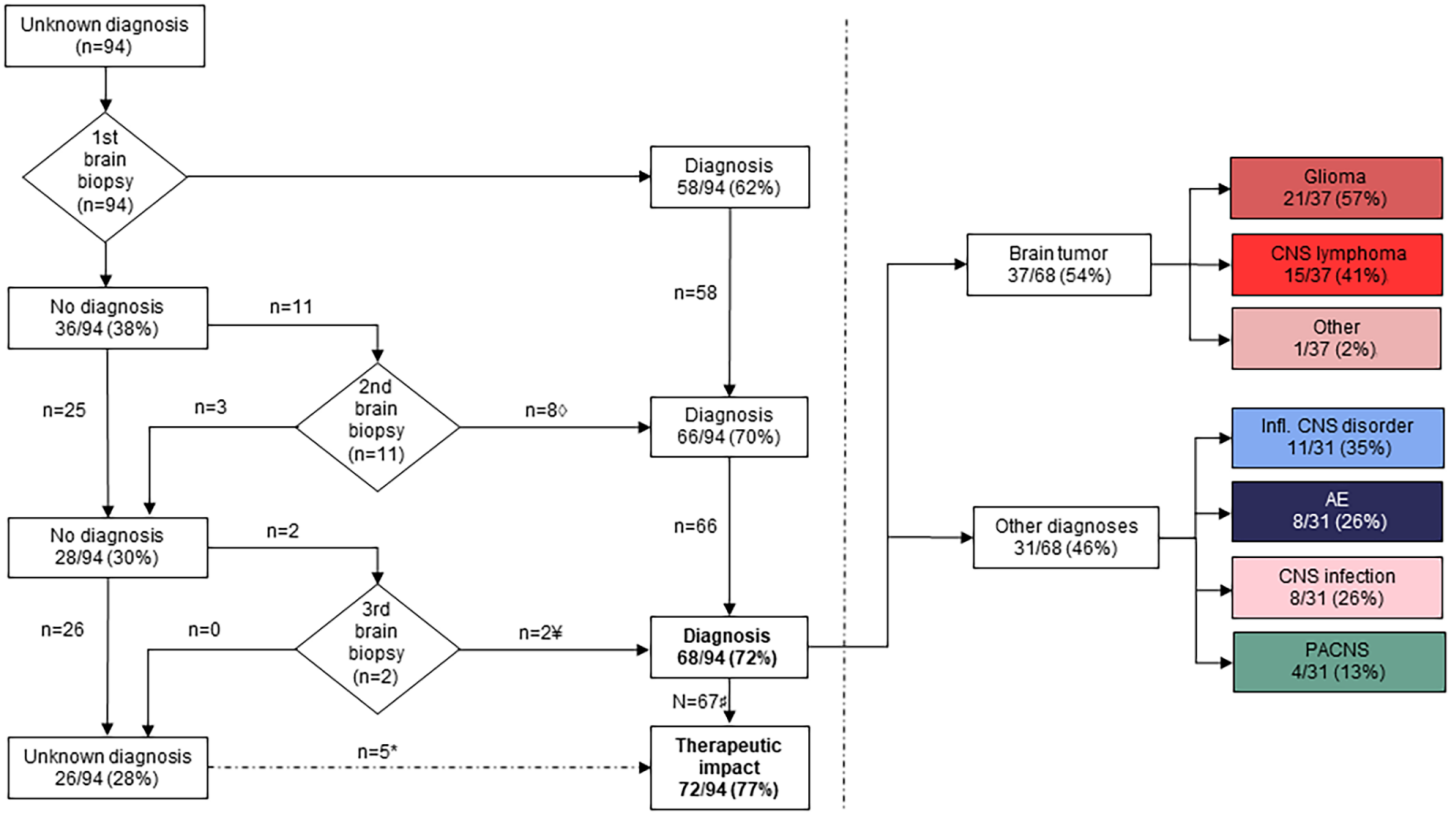
Flowchart showing diagnostic impact of brain biopsy in different subcategories. AE, autoimmune encephalitis; CNS, central nervous system; Infl., inflammatory; PACNS, primary angiitis of the CNS. ◊ 5 gliomas, 3 PCNSL. ¥ 2 gliomas. # One patient deceased shortly after biopsy. *Brain biopsies with therapeutic impact, despite no diagnostic impact. Prebiopsy working diagnosis of neuroinflammatory disorder (*n* = 5), treatment with immunotherapy after exclusion alternative diagnoses by brain biopsy.

**Figure 2:**
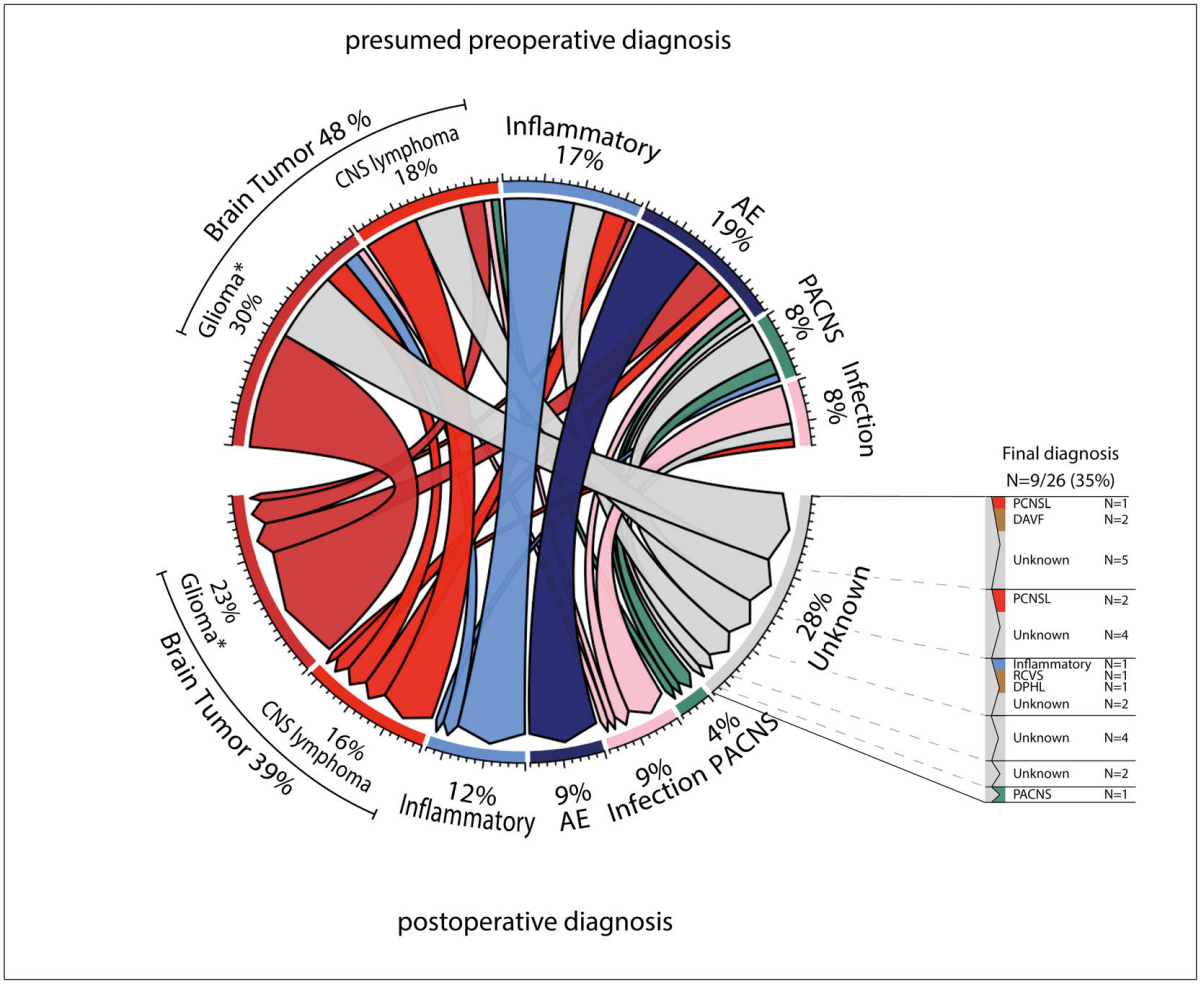
Chord diagram demonstrating change of diagnosis before and after brain biopsy. AE, autoimmune encephalitis; CNS, central nervous system; DAVF, dural arteriovenous fistulas; DPHL, delayed post‐hypoxic leukoencephalopathy; PACNS, primary angiitis of the CNS; PCNSL, primary CNS lymphoma; RCVS, reversible cerebral vasoconstrictive syndrome. *One germ‐cell tumor was included in the glioma category in this figure.

Brain biopsy was diagnostic in 68 of 94 patients (72%) and had therapeutic impact in 72 of 94 patients (77%; Fig. 1). Postoperative diagnostic categories included brain tumors (37/94; 39%), inflammatory CNS disorders (11/94, 12%), AE (8/94, 9%), CNS infections (8/94, 9%), and primary angiitis of the CNS (PACNS; 4/94, 4%; Fig. 2, Table S1). After brain biopsy, preoperative working diagnosis changed in 28 of 94 patients (30%; Fig. 2). Twenty‐six of 94 (28%) patients remained undiagnosed after brain biopsy, of whom 9 were resolved during follow‐up (Fig. 2). Patients with a diagnostic brain biopsy more frequently showed mesiotemporal lesions on brain MRI (31% vs. 8%; *P* = 0.019; Table S2), of whom most patients had a brain tumor (11/21; 52%) or AE (4/21; 19%).

### Brain tumors

Diagnostic yield of brain biopsy was 37 of 40 (93%) in brain tumors. Three patients with a non‐diagnostic brain biopsy were postoperatively diagnosed with a primary CNS lymphoma (PCNSL) by CSF flow cytometry immunophenotyping (FCI; Fig. 2, Table S3). Thirty‐six out of 37 (97%) patients with a diagnostic brain biopsy had a primary brain tumor and 1 patient a systemic lymphoma (Fig. 1). Therapeutic impact was observed in 36 of 37 (97%) patients, consisting of cancer treatment (78%) and best supportive care (22%). One patient unexpectedly deceased prior to initiation of treatment. Eleven of 37 (30%) brain tumor patients had a non‐neoplastic preoperative working diagnosis, of whom most patients (6/11; 55%) were initially suspected of AE (Fig. 2). Compared to matched brain tumor patients from the typical cohort, brain tumor patients from the atypical cohort more often were known with autoimmune diseases (30% vs. 11%; *P* = 0.004) and an immunocompromised status (10% vs. 2%; *P* = 0.035; Table S4). In addition, seizures and brainstem symptoms were more common (43% vs. 21%; *P* = 0.007 and 15% vs. 4%; *P* = 0.019), as opposed to working memory deficits (33% vs. 53%; *P* = 0.022). On brain MRI, bilateral mesiotemporal lesions were more common (10% vs. 0%; *P* < 0.001), in contrast to midline shift (3% vs. 38%; *P* < 0.001), subcortical lesions (55% vs. 82%; *P* < 0.001), and contrast enhancement (80% vs. 91%; *P* = 0.027). Lastly, median duration between onset of symptoms and first brain biopsy was longer in brain tumor patients from the atypical cohort (9 weeks vs. 4 weeks; *P* = 0.011).

**Figure 3:**
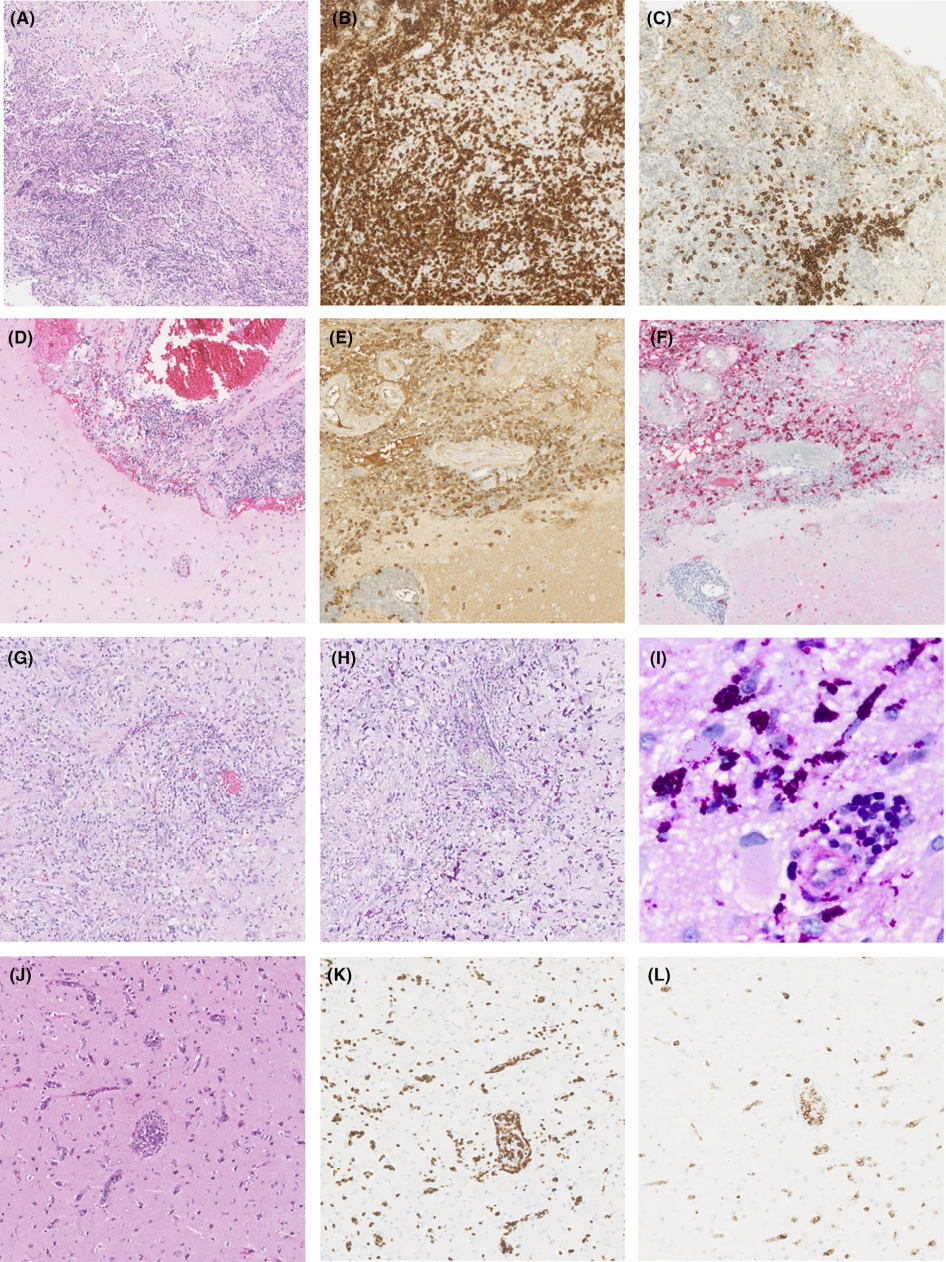
Neuropathological findings of brain biopsy in unexplained CNS disorders. (A–C) Chronic lymphocytic inflammation with pontine perivascular enhancement responsive to steroids (CLIPPERS); Massive parenchymal infiltration (A), predominantly CD3‐positive T cells (B) and some admixture of CD20‐positive B cells (C). Molecular analysis did not show monoclonal T‐cell receptor or B‐cell receptor rearrangements. (D–F) IgG4‐related disease (IgG4‐RD); arachnoid and brain infiltration with a plasma cell‐rich lymphocytic infiltration (D). IgG (E) and IgG4 (F) staining show increased numbers of IgG4‐positive plasma cells and an IgG4/IgG ratio of 0.59. (G–I) central nervous system Whipple disease; clear parenchymal infiltration (G) of PAS‐positive macrophages (H, I). Specific PCR confirmed the presence of Tropheryma whipplei DNA. (J–L) Intravascular T‐cell lymphoma; intravascular and tissue infiltration (J) by CD3 (K) and CD30‐positive T cells (L).

Thirteen repeat biopsies were performed in 11 patients, providing a brain tumor diagnosis of in 10 of 11 (91%) patients (Fig. 1, Table S5). Second and third brain biopsy was diagnostic in 8 of 11 (73%) and 2 of 2 (100%) patients, respectively, increasing overall diagnostic yield of brain biopsy from 62% to 72% (Fig. 1). In 3 of 14 (21%) non‐diagnostic biopsies, performed in 2 patients, off‐target sampling was confirmed by postoperative neuroimaging. Other possible explanations for absence of diagnostic impact included sampling error (11/14; 79%) and preoperative corticosteroid treatment in PCNSL (4/11; 36%). Molecular analysis was performed on first brain biopsy in 3 of 7 (43%) glioma patients, of whom 2 patients (66%) with normal or inconclusive molecular results on first biopsy, analysis on second biopsy revealed diagnostic mutations. In 2 patients, molecular analysis was only applied on second brain biopsy and detected mutations diagnostic of glioma, which were in retrospect also present in the first brain biopsy (Fig. 3). In two patients no molecular analysis was performed, as they were evaluated before NGS implementation (<2013). Main differences between first and diagnostic brain biopsy included selection of alternative target (5/10; 50%), larger (≥5 mm) targeted lesion size (4/10; 40%), open biopsy instead of stereotactic biopsy (4/10; 40%), and discontinuation of corticosteroids (1/10; 10%).

### Non‐neoplastic diagnoses

In non‐neoplastic diagnoses, diagnostic yield and therapeutic impact of brain biopsy were 31 of 54 (57%) and 36 of 54 (67%), respectively. Five non‐diagnostic brain biopsies were considered therapeutically impactful, as an inflammatory disorder was still suspected and immunotherapy could be initiated after exclusion of alternative diagnoses (Fig. 1). Patients with a diagnostic brain biopsy presented more often with working memory deficits (65% vs. 30%; *P* = 0.013) and behavioral symptoms (55% vs. 22%; *P* = 0.014) and more often had ≥2 of the most common symptoms that were observed in non‐neoplastic diagnoses (i.e., focal deficits, working memory deficits, behavioral symptoms, new onset seizures; 48% vs. 4%; *P* = 0.012; Table S6), compared to patients with a non‐diagnostic brain biopsy. On brain MRI, mesiotemporal lesions (uni‐ and bilateral combined and bilateral only) were more common (32% vs. 9%; *P* = 0.039 and 16% vs. 0%; *P* = 0.043), as well as CSF pleocytosis and unique oligoclonal bands (68% vs. 37%; *P* = 0.036 and 36% vs. 7%; *P* = 0.039). In addition, patients with a diagnostic brain biopsy had a longer median duration between onset of symptoms and brain biopsy (17 vs. 7 weeks; *P* = 0.028).

### Inflammatory CNS disorders

Eleven of 31 (35%) non‐neoplastic diagnoses were inflammatory CNS disorders, including 6 of 11 (55%) primary inflammatory CNS disorders and 5 of 11 (45%) systemic inflammatory disorders (Fig. 3, Table S7). Eight of 11 patients (73%) were already suspected of an inflammatory CNS disorder prior to brain biopsy, whereas all patients had one or more characteristics directing to an inflammatory etiology, including signs consistent with a systemic inflammatory disorder (5/11; 45%), suggestive brain MRI abnormalities (5/11; 45%), or inflammatory CSF profile (4/11; 36%; Fig. 2, Table S7). Suggestive MRI abnormalities included pachymeningeal enhancement in IgG4‐related disease (IgG4‐RD; *n* = 2), pontine perivascular enhancement in chronic lymphocytic inflammation with pontine perivascular enhancement responsive to steroids (CLIPPERS; *n* = 2) and an ill‐defined enhancing space occupying lesion in tumefactive multiple sclerosis (*n* = 1). Three of 11 patients (27%) showed strong evidence of a neuroinflammatory disorder, though formal criteria for a specific neuroinflammatory disorder were not met. After demonstration of inflammatory abnormalities and exclusion of alternative causes by brain biopsy, these patients were classified as probable neuroinflammatory disorder (PNID), allowing the initiation of immunotherapy, as described previously.^25^ Therapeutic impact of brain biopsy was observed in all inflammatory CNS disorders, consisting of initiation or escalation of immunotherapy.

### Autoimmune encephalitis

Eight of 18 (44%) suspected AE cases were confirmed by brain biopsy (Fig. 2). In one patient diagnosed with anti‐NMDA receptor encephalitis (autoantibody confirmed), brain biopsy from an enhancing dural lesion was performed, which demonstrated comorbid CNS vasculitis. Treatment regimen was adjusted, as cyclophosphamide was initiated, in addition to first‐line immunotherapy. A diagnosis of seronegative AE (SN‐AE) was established in 7 of 8 (88%) AE patients, of whom 6 of 7 (86%) preoperatively fulfilled the criteria for probable SN‐AE.^3^ In all patients, brain biopsy was performed to exclude alternative diagnoses prior to initiation of long‐term immunotherapy. In particular, differential diagnostic considerations were opportunistic infections due to an immunocompromised status and brain tumors based on atypical features on brain MRI. Perivascular inflammatory infiltrates affecting brain parenchyma without signs of other diagnoses were observed in all patients. After brain biopsy, all 7 patients fulfilled criteria for probable SN‐AE and long‐term immunotherapy was initiated. An alternative diagnosis was obtained in 10 of 18 (56%) patients with suspicion of AE (Fig. 2). Eight of 10 (80%) patients with an AE mimic showed mesiotemporal lesions, of whom all had additional features considered atypical for AE, including persistent enhancement and involvement of extralimbic structures (both 6/8; 75%). Two of 10 AE mimic fulfilled criteria for probable SN‐AE.

### Other diagnoses

In the non‐neoplastic group, 8 of 31 patients (28%) were diagnosed with a CNS infection, of whom 5 of 8 (63%) were opportunistic infections in immunocompromised patients and one patient was diagnosed with HIV encephalopathy (Table S1). Two immunocompetent patients were diagnosed with CNS Whipple's disease (Fig. 3). PACNS was diagnosed by brain biopsy in 4 of 31 patients (13%). In 2 of 8 (25%) patients with suspicion of PACNS, brain biopsy confirmed this diagnosis, while 5 of 8 (63%) remained unexplained and one patient was classified as inflammatory CNS disorder (Fig. 2). The other two PACNS patients originated from other diagnostic categories. A histological diagnosis compatible with vasculitis was obtained in all patients, showing transmural inflammation of the vessel walls. After brain biopsy, all PACNS patients were treated with corticosteroids and cyclophosphamide.

### Non‐diagnostic brain biopsies

Brain biopsy was non‐diagnostic in 26 of 94 (28%) patients, showing normal brain tissue (2/26; 8%) or non‐specific reactive changes (8/26; 31%; Fig. S2). In 16 of 26 (61%) biopsies, mild and non‐specific inflammatory abnormalities were observed, being insufficient to establish a diagnosis in combination with the clinical presentation and other ancillary testing (i.e., brain MRI and CSF testing). Assumed reason for neuropathological misdiagnosis included suspected sampling error (20/26; 77%), preoperative administration of corticosteroids (5/26; 19%), inappropriate indication of brain biopsy (4/26; 15%), and off‐target sampling (2/26%; 8%). Molecular analysis of glioma was performed in 6 of 26 (25%).

### Complications

Complications were observed in 27 of 107 (25%) brain biopsies (Table 3). Twenty‐two (21%) complications were symptomatic, including 4 of 107 (4%) symptomatic hemorrhages. No difference was observed in the occurrence of complications between brain tumors and non‐neoplastic diagnoses (25% vs. 26%; *P* = 0.82). Treatments of symptomatic complications (Grade 2) included initiation or adjustment of antiseizure medication (seizures; *n* = 2), antibiotics (wound infection; *n* = 2), corticosteroids (cerebral edema; *n* = 1), and reversal of anticoagulation (subdural hematoma; *n* = 1; Table S8). Postoperative death within 30 days (Grade 4) occurred in 3 of 107 (3%) brain biopsies, although death was considered clearly biopsy‐related in only 1 of 107 (1%) biopsies, concerning a patient with a massive intracranial hemorrhage 5 days postoperatively (Table S9). In one case, death was considered not biopsy‐related, in view of the long duration between surgical procedure and death (29 days), whereas a direct relation between brain biopsy and death could not be ruled out in the remaining patient. In patients with major complications (grade ≥2), median preoperative modified Rankin scale (mRS) was higher, compared to patients with no or minor complications (grade ≤ 1b; 4 vs. 3 *P* = 0.048; Table S10). No differences were observed between patients with symptomatic intracranial hemorrhages compared to patients with no symptomatic intracranial hemorrhages (Table S11). In repeat brain biopsies, only minor complications were observed, showing a comparable frequency to first brain biopsies (38% vs. 29%; *P* = 0.42).

**Table 3:** Complications of brain biopsy: brain tumors versus non‐neoplastic diagnoses.

Complication grade	All biopsies (*n* = 107)	Biopsies in brain tumors (*n* = 53)	Biopsies in non‐neoplastic diagnoses (*n* = 54)	*P*‐value
1A: Asymptomatic	5 (5%)	3 (6%)	2 (4%)	0.49
1B: Symptomatic, no treatment required^a^	13 (12%)	7 (13%)	6 (11%)	0.74
2: Symptomatic, treatment required^a^	6 (6%)	2 (4%)	4 (7%)	0.35
3: Persistent neurological deficit >6 months	0 (0%)	0 (0%)	0 (0%)	NA
4: Postoperative death within 30 days^b^	3 (3%)	1 (2%)	2 (4%)	0.51
All complications				
Symptomatic	27 (25%)	13 (25%)	14 (26%)	0.87
hemorrhage	4 (4%)	1 (2%)	3 (6%)	0.32

^a^ Described in more detail in Table S8.

^b^ Described in more detail in Table S9.

## Discussion

In this study, we examined the clinical impact and safety of brain biopsy in unexplained CNS disorders. We show that diagnostic yield and therapeutic impact were high (72% and 77%), comparable to previous research,^16^, ^26^, ^27^ while safety was comparable to biopsies in brain tumors.^13^ We found a relatively high percentage of gliomas (20%), which is probably explained by the high number of atypical gliomas at our center, as we are a referral center for neuro‐oncology. Non‐neoplastic categories included inflammatory CNS disorders, AE, CNS infections, and PACNS. After exclusion of brain tumors, diagnostic yield of brain biopsy was 57%. We show that brain biopsy is useful in selected patients with unexplained CNS disorders, as clinical impact is high.

We identified various characteristics useful in establishing a correct brain biopsy indication. Compared to patients with a non‐diagnostic brain biopsy, patients with a non‐neoplastic diagnosis had more symptoms, while mesiotemporal lesions and an inflammatory CSF profile were more common, implicating that the clinical syndrome was more complete. This is particularly relevant in neuroinflammatory disorders, because histological findings are usually insufficient for a definite diagnosis and diagnostic criteria require additional clinical characteristics.^3^, ^20^, ^21^ High preoperative suspicion probably also explains the relative long duration until brain biopsy we observed, as empirical immunotherapy is often initiated before brain biopsy in suspected neuroinflammatory disorders.

Neuroinflammatory disorders represented the large majority (~75%) of non‐neoplastic diagnoses, mostly showing aspecific inflammatory abnormalities in brain biopsy. Nevertheless, clinical impact of brain biopsy was high by allowing safe initiation or escalation of immunotherapy by excluding alternative diagnoses. In suspected systemic inflammatory disorders, brain biopsy was performed if no systemic lesions were present or if deviation of expected disease course was observed. In our study, an alternative diagnosis was obtained in ~35% of suspected neuroinflammatory disorders, including infections and lymphomas. We demonstrate the utility of brain biopsy in suspected neuroinflammatory disorders and emphasize that corticosteroid maintenance treatment should be avoided until a definite diagnosis is established, particularly if an infection or lymphoma is among the differential diagnosis.^28^, ^29^

Most CNS infections occurred in immunocompromised patients, showing the relevance of brain biopsy in this category.^8^, ^16^ CNS Whipple's disease was exceptional in this category, as it was the only CNS infection in immunocompetent patients.^30^ In this study, mNGS was applied on a selected subset of brain biopsies, as this technique is currently not yet part of routine diagnostics. However, standardized application of mNGS might increase diagnostic yield of brain biopsy in the future.^31^

AE represented a unique category, as its diagnosis strongly relies on autoantibody testing. Not unexpectedly, SN‐AE was the most common subtype (~90%). Its diagnosis is based on the 2016 clinical AE criteria, which require a rapidly progressive neuropsychiatric syndrome and ≥2 of the following features: MRI suggestive of AE, inflammatory CSF profile or brain biopsy showing inflammation.^3^ Previously, we showed that these criteria are highly specific (>95%), though clinicians should always be aware of AE mimics.^25^ In this study, only ~40% of suspected SN‐AE could be confirmed. All biopsy confirmed SN‐AE patients had a high preoperative suspicion (86% fulfilled SN‐AE criteria), atypical characteristics introducing diagnostic uncertainty and indication for long‐term or second‐line immunotherapy. Only 20% of AE mimics fulfilled SN‐AE criteria, implicating preoperative suspicion was relatively low. In addition, 80% of AE mimics showed atypical mesiotemporal lesions on brain MRI, which we identified earlier as important diagnostic pitfall in AE misdiagnosis.^25^ In summary, brain biopsy is relevant in selected cases of suspected AE, in particular if long‐term immunotherapy is planned and atypical radiological features are present.

We show that brain tumors had a high occurrence of atypical features. A midline shift was extremely rare, indicating limited mass effect. In addition, brainstem lesions and non‐enhancing lesions were relatively common, which are rarer presentations of adult brain tumors.^32^, ^33^ Interestingly, duration between symptom onset and first brain biopsy was relatively long, possibly reflecting both slow disease progression and diagnostic uncertainty. Nevertheless, diagnostic yield of biopsy in brain tumors was high (~95%), as described earlier.^6^ Although we cannot completely rule out brain tumor diagnoses in patients with unknown diagnoses, this was considered highly unlikely in most patients, in view of absent clinico‐radiological progression during follow‐up. Two cases of PCNSL were postoperatively identified by CSF FCI, demonstrating that this (repeated) investigation should always be part of the diagnostic work‐up in unexplained CNS disorders.^34^ Our findings emphasize that brain tumors are relevant to include in the differential diagnosis of unexplained CNS disorders, even if features atypical for tumors are present.

The occurrence of brain biopsy‐related complications did not differ between brain tumors and non‐neoplastic diagnoses. Intracranial hemorrhage occurred in 4% of brain biopsies, which was comparable to earlier studies.^13^, ^35^ However, the frequency of major complications was higher than reported earlier,^16^ which is probably explained by the high number of critically ill patients in this study, as 15% of patients had mRS 5 and median mRS was higher in patients with major complications. This is supported by previous research, showing that mortality after brain biopsy is higher in critically ill patients.^36^ Three patients died within 30 days after brain biopsy. A clear relation could only be established in one brain biopsy, which was performed as last resort in a critically ill patient, being not representative for the total cohort. This emphasizes that severe complications particularly occur only in a selection of patients with an increased risk profile and complication severity is strongly related to disease severity.^36^ Our findings show that brain biopsy in unexplained CNS disorders is relatively safe, though preoperative estimation of risk/benefit ratio is essential, as major complications might occur, especially in the critically ill patient.

In 28% of patients, no diagnosis could be established by brain biopsy. Notably, non‐specific inflammatory alterations were observed in more than half of these patients, which were insufficient to provide a specific diagnosis, as clinical criteria for neuro‐inflammatory disorders were not met. However, brain biopsy was still impactful in a subset of patients' suspicion of a neuro‐inflammatory disorder, as it altered treatment strategy (i.e., initiation of immunotherapy) by excluding other diagnoses. Importantly, in ~20% of brain biopsies showing inflammatory abnormalities, a brain tumor was identified during follow‐up, demonstrating the complexity of its interpretation, as these features might also be present in other diseases.^15^, ^37^ Our findings demonstrate the clinical impact of brain biopsy in a specific category of patients not satisfying existing diagnostic criteria, though cautious and multidisciplinary interpretation of neuropathological findings is strongly recommended.

We found that in non‐neoplastic diagnoses, the cortex was more often targeted in diagnostic brain biopsies and, nearly significant, open biopsy was more frequently performed. Although both differences probably reflect the same phenomena, as brain biopsy technique was directed by lesion characteristics, this might suggest that in diffuse, cortical lesions, diagnostic yield is higher in open biopsy. Overlying leptomeninges should always be included, as these structures are often affected in inflammatory and infectious diseases. Importantly, no difference was observed in complication rates between different biopsy techniques. Larger studies are needed to confirm this hypothesis.

A relatively high percentage of brain tumors (25%) was diagnosed after repeat biopsy. Diagnostic yield of repeat biopsy was high (~90%) and showed a similar safety profile as first brain biopsies, as reported earlier.^38^, ^39^ Various characteristics contributed to the high success rate of repeat biopsies. First, two biopsies could have been avoided by molecular analysis on initial, non‐diagnostic biopsies. In previous research, molecular analysis provided a diagnosis in 61%–77% of cases with an inconclusive histological diagnosis.^19^, ^40^ Therefore, it is strongly recommended to first apply molecular analysis on a non‐diagnostic brain biopsy. However, avoidance of repeat biopsies by application of molecular analysis should be weighted carefully and established on reliable techniques (i.e., strict cut‐off values), in order to prevent false‐positive results and subsequent misdiagnoses.^19^ Second, in diagnostic repeat biopsies, open biopsy was performed more often and larger lesions were targeted, which are both associated with a higher diagnostic yield.^39^, ^40^ Lastly, cessation of corticosteroids might have contributed to the diagnosis of PCNSL, although inconsistent results are reported in literature.^41^, ^42^ Our findings implicate that the added value of repeat brain biopsy is high when diagnostic uncertainty persists after first biopsy, although molecular analysis on first biopsy should always be considered first.

This study has some limitations. First, no pediatric patients were included. Therefore, our findings cannot be applied to this patient category. Pediatric studies describing this topic are scarce,^43^ emphasizing the need for studies in this population. Second, due to the heterogeneity of non‐neoplastic diagnoses, we were possibly unable to detect relevant differences. However, by comprehensively describing clinical profiles of different categories, we provide a clear overview of relevant items that should be taken into consideration prior to brain biopsy. Lastly, only lesional brain biopsies were performed in this study. Therefore, our results are not applicable to patients without a radiological intracranial lesion.

In summary, the clinical impact of brain biopsy in unexplained CNS disorders is high, by allowing crucial treatment decisions in the large majority of a highly challenging population. In addition, major complications are relatively rare, despite the high presentation of critically ill patients. In case a diagnosis cannot be established by non‐invasive diagnostics, brain biopsy should particularly be considered in the following conditions: (1) suspicion of brain tumor (2) high suspicion of a neuroinflammatory disorder with indication for long‐term or second‐line immunotherapy, and (3) CNS lesion in immunocompromised patients. A multidisciplinary approach is essential to obtain optimal clinical impact. Further research should focus on the characterization of specific pathological features of neuroinflammatory disorders and optimization of mNGS techniques in CNS infections.

## Author Contributions

R.W.v.S., S.S., J.M.d.V., R.B., and M.T. contributed to the conception and design of the study. All authors contributed to acquisition and analysis of data. R.W.v.S., S.S., J.M.d.V., R.B., and M.T. contributed to drafting the text and preparing the figures.

## Funding Information

This study has received funding from ZonMw (VIMP program; 2022).

## Conflicts of Interest

Nothing to report.

## Supporting information


Data S1.
**Figure S1.** Flowchart of patient inclusion.
**Figure S2.** Flowchart showing main characteristics of non‐diagnostic brain biopsies.
**Table S1.** Specific diagnoses based on brain biopsy per diagnostic subcategory.
**Table S2.** Comparison of patients with brain biopsy with diagnostic impact vs. no diagnostic impact (all patients).
**Table S3.** Primary CNS lymphomas identified during follow‐up (non‐diagnostic brain biopsy).
**Table S4.** Comparison of patients with brain tumors; atypical vs. typical presentation (1:3).
**Table S5.** Description of patients with repeat brain biopsies.
**Table S6.** Comparison of patients with brain biopsy with diagnostic impact vs. no diagnostic impact in non‐neoplastic diagnoses.
**Table S7.** Inflammatory CNS disorders.
**Table S8.** Overview of grade 1B and 2 complications.
**Table S9.** Description of patients with grade 4 complication (postoperative death within 30 days).
**Table S10.** Comparison of patients with no or minor (grade ≤1b) vs. major (grade ≥2) complications.
**Table S11.** Comparison of patients with no symptomatic intracranial hemorrhage vs. symptomatic intracranial hemorrhage.

## Data Availability

Anonymized study data will be shared pending review of a request to the corresponding author from qualified individuals.
