# Supplementary material for: Clinical impact and safety of brain biopsy in unexplained central nervous system disorders: a real‐world cohort study
Source: Ann Clin Transl Neurol. 2025 Feb 21;12(4):792–804. doi: 10.1002/acn3.70000 (PMC12040505; doi:10.1002/acn3.70000)
Supplement: Supplementary file 1 — Data S1. Figure S1. Flowchart of patient inclusion. Figure S2. Flowchart showing main characteristics of non‐diagnostic brain biopsies. Table S1. Specific diagnoses based on brain biopsy per diagnostic subcategory. Table S2. Comparison of patients with brain biopsy with diagnostic impact vs. no diagnostic impact (all patients). Table S3. Primary CNS lymphomas identified during follow‐up (non‐diagnostic brain biopsy). Table S4. Comparison of patients with brain tumors; atypical vs. typical presentation (1:3). Table S5. Description of patients with repeat brain biopsies. Table S6. Comparison of patients with brain biopsy with diagnostic impact vs. no diagnostic impact in non‐neoplastic diagnoses. Table S7. Inflammatory CNS disorders. Table S8. Overview of grade 1B and 2 complications. Table S9. Description of patients with grade 4 complication (postoperative death within 30 days). Table S10. Comparison of patients with no or minor (grade ≤1b) vs. major (grade ≥2) complications. Table S11. Comparison of patients with no symptomatic intracranial hemorrhage vs. symptomatic intracranial hemorrhage. [file ACN3-12-792-s001.docx]

**Supplementary Material**

**Clinical impact and safety of brain biopsy in unexplained central nervous system disorders: a real-world cohort study.**

**List of Supplements: 1 Supplementary Figure and 9 Supplementary Tables**

**Supplementary Figures**

eFigure 1: Flowchart of patient inclusion
eFigure 2: Flowchart showing main characteristics of non-diagnostic brain biopsies

**Supplementary Tables**eTable 1: Specific diagnoses based on brain biopsy per diagnostic subcategory

eTable 2: Comparison of patients with brain biopsy with diagnostic impact vs. no diagnostic impact (all patients)

eTable 3: Primary CNS lymphomas identified during follow-up (non-diagnostic brain biopsy)
eTable 4: Comparison of patients with brain tumors; atypical vs. typical presentation (1:3)

eTable 5: Description of patients with repeat brain biopsies

eTable 6. Comparison of patients with brain biopsy with diagnostic impact vs. no diagnostic impact in non-neoplastic diagnoses

eTable 7: Inflammatory CNS disorders

eTable 8: Overview of grade 1B and 2 complications

eTable 9: Description of patients with grade 4 complication (postoperative death within 30 days)

eTable 10: Comparison of patients with no or minor (grade ≤1b) vs. major (grade ≥2) complications
eTable 11: Comparison of patients with no symptomatic intracranial hemorrhage vs. symptomatic intracranial hemorrhage

**eFigure 1: Flowchart of patient inclusion**

**
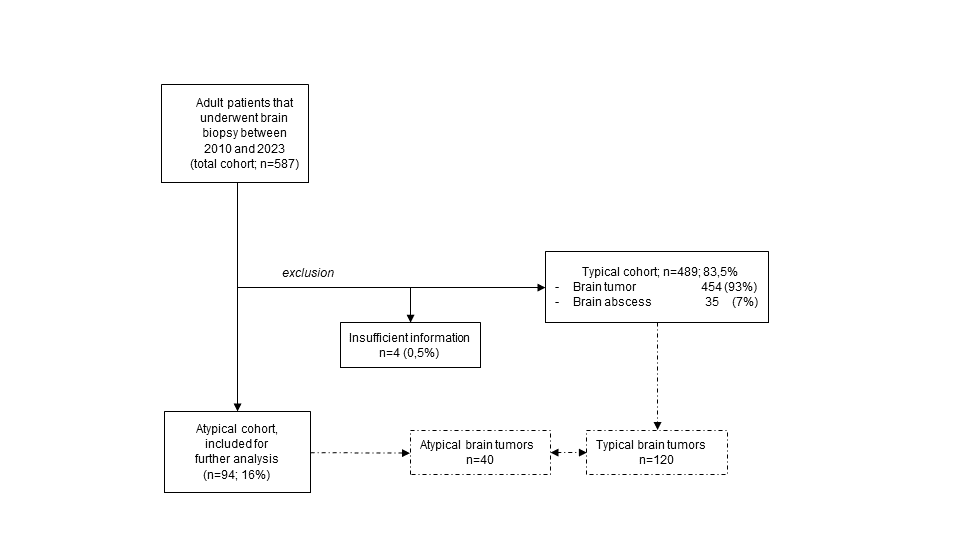
**

**eFigure 2: Flowchart showing main characteristics of non-diagnostic brain biopsies**

**
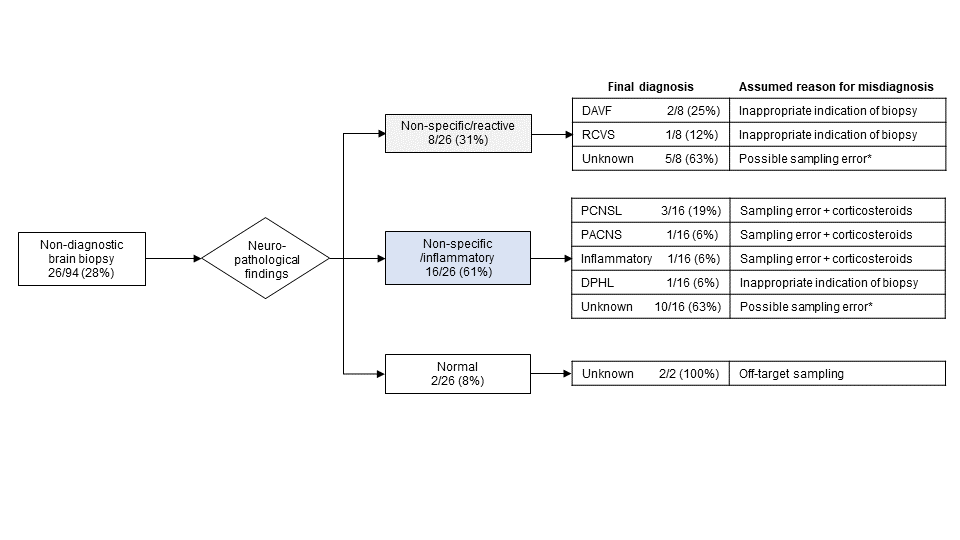
**

*Labeled as possible sampling error, as no final diagnosis was obtained during follow-up. DAVF=dural arteriovenous fistula. DPHL=delayed post-hypoxic leukoencephalopathy. PACNS=primary angiitis of the central nervous system. PCNSL=primary cerebral nervous system lymphoma. RCVS=reversible cerebral vasoconstrictive syndrome.

**eTable 1: Specific diagnoses based on brain biopsy per diagnostic subcategory.**

| **Diagnostic subcategory** | **Specific diagnoses**  **(n=68)** |  |
| --- | --- | --- |
| Brain tumors | No. (%) | 37 (54) |
|  | Grade IV glioma  Grade II/III glioma  B cell lymphoma  T cell lymphoma  Non-germinomatous germ cell tumor | 17 (46) 4 (11) 11 (30) 4 (11) 1 (2) |
| CNS inflammatory disorder | No. (%) | 11 (16) |
|  | Probable neuroinflammatory disorder (PNID)  IgG4 related disease(IgG4-RD)  Chronic lymphocytic inflammation with pontine perivascular enhancement  responsive to steroids (CLIPPERS)  Neurosarcoidosis  Reumatoid meningitis  Tumefactive multiple sclerosis (MS) | 3 (27) 2 (18)  2 (18) 2 (18) 1 (9) 1 (9) |
| Autoimmune encephalitis | No. (%) | 8 (12) |
|  | Seronegative autoimmune encephalitis  Anti-NMDAR encephalitis with comorbid CNS vasculitis | 7 (88) 1 (12) |
| CNS infection | No. (%) | 8 (12) |
|  | Progressive multifocal leukoencephalopathy (PML)*  CNS Whipple’s disease  Cerebral yeast infection*  HIV encephalopathy  Cerebral toxoplasmosis* | 3 (38) 2 (25) 1 (12) 1 (12) 1 (12) |
| CNS vasculitis | No. (%) | 4 (6) |
|  | Primary angitis of the CNS (PACNS) | 4 (100) |

CNS=central nervous system. HIV=human immunodeficiency virus. NMDAR=NMDA receptor. *Immunocompromised status.

**eTable 2: Comparison of patients with brain biopsy with diagnostic impact vs. no diagnostic impact (all patients).**

|  | **Diagnostic impact**  **N=68** | **No diagnostic impact**  **N=26** | **P-value** |
| --- | --- | --- | --- |
| Female gender no. (%) | 27 (40) | 12 (46) | 0.22 |
| Age at biopsy in years (median; IQR; range) | 59; 52-69; 19-79 | 54; 43-72; 30-79 | 0.92 |
| Past medical history no. (%)  autoimmune disease   systemic malignancy  [immunocompromised](https://www.google.com/search?sca_esv=569142147&rlz=1C1GCEJ_en&q=immunocompromised&spell=1&sa=X&ved=2ahUKEwjc1LWTss2BAxWwxQIHHTa7D90QkeECKAB6BAgGEAE) status | 18 (27) 10 (15)  15 (22) | 10 (39) 4 (14)  4 (15) | 0.26 0.93 0.47 |
| Initial presentation. (%)  subacute onset   focal deficits  working memory deficits  behavioral symptoms  new-onset seizures  impaired consciousness  cerebellar ataxia  brainstem symptoms | 63 (93)  36 (53)  32 (47) 29 (43)  25 (37)  10 (15) 11 (16)  11 (16) | 24 (92) 12 (46)  8 (31)  7 (27) 8 (31)  6 (23) 3 (12)  1 (4) | 0.96 0.56  0.15 0.16 0.59 0.33 0.57 0.11 |
| MRI brain performed no. (%)  cortical  mesiotemporal  unilateral  bilateral  subcortical and white matter  deep gray matter  brainstem  cerebellum  meninges  increased T2/FLAIR signal  enhancement  restricted diffusion | 67 (99) 46 (68) 21 (31)  12 (18)  9 (13) 35 (52)  17 (25)  19 (28)  10 (15)  13 (19)  62 (93)  51 (75)  21/67 (31) | 26 (100) 13 (50) 2 (8)  2 (8)  0 (0) 71 (69) 11 (42)  3 (12)  2 (8) 5 (19) 22 (85)  22 (85)  7 (27) | 0.53 0.11  **0.019**  0.23  0.051  0.12 0.10 0.09  0.29 0.99 0.29  0.48  0.76 |
| CSF performed no. (%)  WBC>5/ul  CSF specific oligoclonal bands | 56 (82) 29/56 (52)  11/38 (29) | 21 (81) 8/21 (38) 2/16 (13) | 0.86 0.37  0.20 |
| EEG performed no. (%)  epileptic abnormalities | 25 (37) 2/25 (8) | 2 (8) 0 (0) | **0.005** 0.87 |
| Systemic features no. (%)  tumor  lymphadenopathy | 0 (0)  9 (13) | 2 (8)  5 (20) | 0.074 0.42 |
| Modified Rankin Scale (mRS; median; IQR; range) | 3; 2-4; 1-5 | 3; 2-4; 2-5 | 0.50 |
| Duration between onset and brain biopsy in weeks* (median; IQR; range) | 14; 6-37; 2-780 | 6; 3-21; 0-216 | 0.25 |
| Preoperative corticosteroids no.▲ (%)  Interval last corticosteroid and biopsy in days (median; IQR; range) | 21 (31)  9; 0-44; 0-59 | 8 (31)  15; 3-23; 0-28 | 1  0.91 |
| Biopsy technique* no. (%)  stereotactic biopsy  open biopsy | 36 (53) 32 (47) | 16 (62) 10 (39) | 0.45 0.45 |
| Target of brain biopsy* no. (%)  cortical  corticomeningeal  subcortical  deep gray matter  brainstem/cerebellum  enhancement | 38 (56)  12/38 (32)  20 (29)  2 (3) 8 (12)  51 (75) | 10 (39) 4/10 (40)  13 (50) 3 (12)  0 (0) 22 (85) | 0.13  0.44  0.061 0.097 0.066 0.32 |

IQR=interquartile range. CSF=cerebrospinal fluid. WBC=white blood cell count. FLAIR=Fluid-Attenuated inversion recovery. *In patients with multiple brain biopsies, the diagnostic biopsy was selected. ▲<60 days prior to brain biopsy.

**eTable 3: Primary CNS lymphomas identified during follow-up (non-diagnostic brain biopsy)**

| **Final  diagnosis** | **Sex;  Age (range)** | **Clinical**  **picture** | **Ancillary**  **testing** | **Cortico-**  **steroids** | **No. brain**  **biopsies** | **Biopsy technique** | **Histological**  **diagnosis** | **Decisive**  **diagnostic test**  **(after brain biopsy)** | **Duration from first brain biopsy until diagnosis (weeks)** |
| --- | --- | --- | --- | --- | --- | --- | --- | --- | --- |
| 1. B cell lymphoma | M;  60-65 | Subacte cognitive decline | MRI: enhancing lesion right frontotemporal.  CSF: not performed.  CT total body: normal. | no | 1 | Stereotactic | Perivascular infiltrates, reactive changes | CSF FCI | 12 |
| 2. B cell lymphoma | F;  75-80 | Dyspha-sia, hemiparesis | MRI: enhancing lesion right frontal and corpus callosum.  CSF: not performed.  PET-CT/CT: not performed. | yes | 1 | Stereotactic | Encephalitis | CSF FCI | 2 |
| 3. B cell lymphoma▲ | M;  70-75 | Dyspha-sia, hemipar-esis | MRI: enhancing lesion left hemisphere.  CSF: mild pleocytosis, FCI normal.  CT total body: normal | yes | 2 | Stereotactic; Stereotactic | Encephalitis;  encephalitis | Mass resection | 76 |

CNS=central nervous system. FCI=flow cytometry immunophenotyping. ▲This patient is also described in eTable 5.

**eTable 4: Comparison of patients with brain tumors; atypical vs. typical presentation (1:3)**

|  | **Brain tumors with atypical presentation**  **N=40** | **Brain tumors with typical presentation**  **N=120** | **P-value** |
| --- | --- | --- | --- |
| Female gender no. (%) | 16 (40) | 48 (40) | 1 |
| Age at biopsy in years (median; IQR; range) | 60; 52-69; 19-79 | 62; 53-69; 38-82 | 0.89 |
| Past medical history no. (%)  autoimmune disease   systemic malignancy  [immunocompromised](https://www.google.com/search?sca_esv=569142147&rlz=1C1GCEJ_en&q=immunocompromised&spell=1&sa=X&ved=2ahUKEwjc1LWTss2BAxWwxQIHHTa7D90QkeECKAB6BAgGEAE) status | 12 (30) 2 (5)  4 (10) | 13 (11)  19 (16)  4 (2) | **0.004**  0.08 **0.035** |
| Initial presentation. (%)  subacute onset   focal deficits  working memory deficits  behavioral symptoms  new-onset seizures  impaired consciousness  cerebellar ataxia  brainstem symptoms | 37 (93)  21 (53)  13 (33)  14 (35)  17 (43)  3 (8)  7 (18)  6 (15) | 105 (88)  73 (61) 64 (53)  25 (20)  25 (21)  5 (4)  13 (11)  6 (5) | 0.39 0.35 **0.022** 0.054 **0.007** 0.32 0.27 **0.038** |
| MRI brain performed no. (%)  cortical  mesiotemporal  unilateral  bilateral  subcortical and white matter  deep gray matter  brainstem  cerebellum  meninges  increased T2/FLAIR signal  enhancement  restricted diffusion  midlineshift | 39 (98)  39 (73)  11 (28)  7 (18)  4 (10)  22 (55)  14 (35)  11 (28)  5 (13)  4 (10)  37 (93)  32 (80) 13/39 (33) 1 (3) | 120 (100)  91 (76)  25 (21)  24 (20)  0 (0)  98 (82)  34 (28)  14 (12)  9 (8) 3 (3)  113 (95)  109 (91)  33 (28)  45 (38) | 0.08  0.67  0.58  0.73  **<0.001 <0.001** 0.56 **0.017**  0.33  0.066 0.26  **0.027** 0.21 **<0.001** |
| EEG performed no. (%)  epileptic abnormalities | 13 (33) 1/13 (8) | 3 (3) 1/3 (33) | **<0.001** 0.35 |
| CSF performed no. (%)  WBC>5/ul  oligoclonal bands | 29 (73)  11/29 (38)  4/17 (24) | 25 (21) 13/25 (52) 0/0 (0) | **<0.001**  0.30 0.21 |
| Systemic features no. (%)  tumor  lymphadenopathy | 0 (0) 3 (8) | 5 (4)  5 (5) | 0.19 0.55 |
| Duration between onset and brain biopsy in weeks (median; IQR; range) | 9; 4-28; 2-139 | 4; 2-15; 0-263 | **0.011** |
| Karnofsky performance scale  (KPS; median; IQR; range) | 80; 60-90; 30-100 | 80; 60-80, 30-100 | 0.85 |

Patients were matched 1:3 for age (+-5 years), gender and tumor type. IQR=interquartile range. WBC=white blood cell count. FLAIR=Fluid-Attenuated inversion recovery.

**eTable 5: Description of patients with repeat brain biopsies**

| **Final  diagnosis** | **Brain biopsy no.; year** | **Sex; age**  **(range)** | **Interval  beween**  **biopsies, days** | **Cortico-**  **steroids** | **Biopsy**  **target** | **Targeted lesion**  **diameter**  **(mm)** | **Biopsy**  **technique** | **Molecular markers**  **of glioma** | **Neuro-pathological  diagnosis** | **Assumed reason**  **for**  **misdiagnosis** |
| --- | --- | --- | --- | --- | --- | --- | --- | --- | --- | --- |
| 1. Glioma-1 | 1/3; 2011◊ | F;  70-75 | NA | no | Right parietal | 14 | stereotactic | not tested | Gliosis | Off-target sampling♯ |
|  | 2/3: 2012 | ‘’ | 31 | no | Right parietal | 17 | stereotactic | not tested | Perivasculair infiltrates | Off-target sampling♯ |
|  | 3/3;  2012 | ‘’ | 46 | no | Right fronto-temporal (alternative lesion) | 28 | open | not tested | Grade IV glioma |  |
| 2. Glioma-2 | 1/3; 2015 | M;  45-50 | NA | no | Right frontal | 7 | stereotactic | normal | Gliosis, calcifications | Sampling error |
|  | 2/3;  2016 | ‘’ | 308 | no | Right frontal | 7 | stereotactic | normal | Perivascular infiltrates | Sampling error |
|  | 3/3;  2016 | ‘’ | 55 | no | Left frontal (alternative lesion) | 10 | stereotactic | inconclusive | Grade IV  Glioma |  |
| 3. Glioma-3 | 1/2; 2015 | M;  65-70 | NA | yes | Left frontal | 33 | Stereotactic | not tested* | Gliosis, necrosis | Sampling error |
|  | 2/2;  2015 | ‘’ | 13 | yes | Left frontal | 33 | open | methylated MGMT gene promoter, PTEN- and TP-53 mutation, imbalance chromosome 7 and 10, partial loss chromosome 19q* | Grade IV glioma |  |
| 4. Glioma-4 | 1/2;  2020 | M;  50-55 | NA | no | Right cerebellar | 49 | Stereotactic | no classifying abnormalities | Gliosis, ischemia | Sampling error |
|  | 2/2; 2020 | ‘’ | 73 | no | Right cerebellar | 49 | Open | IDHwt, MSH2 mutation | Astrocytic tumor |  |
| 5. Glioma-5 | 1/2;  2011◊ | M;  70-75 | NA | no | Right parietal | 27 | Stereotactic | not tested | necrosis | Sampling error |
|  | 2/2; 2011 | ‘’ | 25 | no | Right parietal | 38 | Stereotactic | not tested | Grade IV glioma |  |
| 6. Glioma-6 | 1/2;  2022 | F;  55-60 | NA | yes | Right hippocapus | Diffuse edema | Open | inconclusive | Reactive changes, T cells | Sampling error |
|  | 2/2; 2022 | ‘’ | 72 | no | Right hippo-campus, insula (alternative lesion) | Diffuse edema | Open | IDHwt, TERT promoter | Grade IV glioma |  |
| 7. Glioma-7 | 1/2;  2017 | F;  45-50 | NA | yes | Left temporal | Diffuse edema | Open | not tested* | Leptomeningitis, encephalitis | Samping error |
|  | 2/2;  2018 | ‘’; 45-50 | 213 | yes | Left temporal | 24 | Open | Methylated MGMT gene promoter, TERT promoter* | Grade IV glioma |  |
| 8. PCNSL-1 | 1/2;  2017 | M; 55-60 | NA | yes | Left cerebellar | 12 | Open | NA | Perivascular infiltrates | Off-target sampling♯, corticosteroids |
|  | 2/2; 2017 | ‘’ | 14 | yes | Left cerebellar | 12 | Open | NA | CNS large B cell lymphoma |  |
| 9. PCNSL-2 | 1/2; 2010 | F;  55-60 | NA | yes | Right parietal | 14 | Stereotactic | NA | Possible demyelination | Sampling error,  corticosteroids |
|  | 2/2; 2010 | ‘’ | 76 | no | Right caudate nucleus (alternative lesion) | 29 | Stereotactic | NA | CNS large B cell lymphoma |  |
| 10. PCNSL-3 | 1/2; 2019 | F; 65-70 | NA | No | Right parietal | 12 | Stereotactic | NA | encephalitis | Sampling error |
|  | 2/2; 2019 | ‘’ | 21 | No | Right occipital (alternative lesion) | 15 | Open | NA | Intracavascular T-cell lymphoma |  |
| 11. Unknown▲ | 1/2; 2012 | M; 70-75 | NA | yes | Left frontal | 34 | stereotactic | NA | Encephalitis | Sampling error, corticosteroids |
|  | 2/2; 2012 | ‘’; 70-75 | 325 | yes | Left frontal | 20 | stereotactic | NA | Encephalitis | Sampling error, corticosteroids |

NA=not applicable. PCNSL=primary central nervous system lymphoma. ◊ Clinical evaluation prior to implementation of molecular analysis of glioma in 2013. *Molecular biomarkers of glioma were retrospectively also abnormal in first brain biopsy. ♯ Confirmed by postbiopsy imaging. ▲Also described in eTable 3, diagnosed with PCNSL after mass resection due to ongoing suspicion of PCNSL.

**eTable 6. Comparison of patients with brain biopsy with diagnostic impact vs. no diagnostic impact in non-neoplastic**

**diagnoses**

|  | **Diagnostic impact**  **N=31** | **No diagnostic impact**  **N=23** | **P-value** |
| --- | --- | --- | --- |
| Female gender no. (%) | 12 (39) | 13 (57) | 0.19 |
| Age at biopsy in years (median; IQR; range) | 60; 52-70; 32-77 | 52; 39-68; 30-79 | 0.39 |
| Past medical history no. (%)  autoimmune disease   systemic malignancy  [immunocompromised](https://www.google.com/search?sca_esv=569142147&rlz=1C1GCEJ_en&q=immunocompromised&spell=1&sa=X&ved=2ahUKEwjc1LWTss2BAxWwxQIHHTa7D90QkeECKAB6BAgGEAE) status | 7 (23) 8 (26) 11 (36) | 9 (39)  4 (17) 4 (17) | 0.19  0.46  0.14 |
| Initial presentation. (%)  subacute onset   focal deficits  working memory deficits  behavioral symptoms  new-onset seizures  impaired consciousness  cerebellar ataxia  brainstem symptoms | 28 (90) 17 (55)  20 (65) 17 (55)  8 (26) 7 (23)  4 (13) 5 (16) | 22 (96) 10 (44) 7 (30)  5 (22)  8 (35) 6 (26)  3 (13)  1 (4) | 0.46 0.41 **0.013** **0.014**  0.48 0.77 0.99 0.17 |
| ≥2/4 most common symptoms* | 10 (48) | 1 (4) | **0.012** |
| MRI brain performed no. (%)  cortical  mesiotemporal  unilateral  bilateral  subcortical and white matter  deep gray matter  brainstem  cerebellum  meninges  increased T2/FLAIR signal  enhancement  restricted diffusion | 34 (100)  20 (65) 10 (32) 5 (16)  5 (16)  15 (48) 5 (16) 8 (26) 5 (16) 9 (29) 29 (94)  22 (71) 10 (32) | 20 (100) 10 (44) 2 (9) 2 (9)  0 (0)  15 (65) 9 (39) 3 (13) 2 (9) 5 (22) 20 (87)  19 (83) 6 (26) | 1  0.12 **0.039** 0.42 **0.043**  0.22 0.056 0.25 0.42 0.55 0.41  0.32 0.62 |
| CSF performed no. (%)  WBC>5/ul  oligoclonal bands | 28 (90)  19/28 (68) 8/22 (36) | 20 (87) 7/20 (35) 1/15 (7) | 0.70 **0.036 0.039** |
| EEG performed no. (%)  epileptic abnormalities | 12 (39) 1 (8) | 2 (9) 0 (0) | **0.013** 0.85 |
| Systemic features no. (%)  tumor  lymphadenopathy | 0 (0)  6 (19) | 2 (9)  5 (23) | 0.094 0.77 |
| Modified Rankin Scale (MRS; median; IQR; range) | 3; 3-4; 2-5 | 3; 2-4; 2-5 | 0.69 |
| Duration between onset and brain biopsy in weeks* (median; IQR; range) | 17; 8-59; 2-780 | 7; 2-418-; 0-216 | **0.028** |
| Preoperative corticosteroids no.▲ (%)  Interval last corticosteroid and biopsy in days (median; IQR; range) | 13 (42)  32; 0-44; 0-54 | 6 (26)  19; 13-25; 10-28 | 0.23  0.069 |
| Biopsy technique no. (%)  stereotactic biopsy  open biopsy | 10 (32) 21 (68) | 13 (57) 10 (44) | 0.075 0.075 |
| Target of brain biopsy no. (%)  cortical  corticomeningeal  subcortical  deep gray matter  brainstem/cerebellum  enhancement | 22 (71)  9/22 (41)  7 (23)  0 (0) 2 (7)  22 (71) | 10 (44)  4/10 (40)  11 (48)  2 (9)  0 (0)  19 (83) | **0.042**  0.64  **0.049**  0.18 0.33  0.32 |

IQR=interquartile range. CSF=cerebrospinal fluid. WBC=white blood cell count. FLAIR=Fluid-Attenuated inversion recovery. *focal deficits, working memory deficits, behavioral symptoms, new-onset seizures. ▲<60 days prior to brain biopsy.

**eTable 7: Inflammatory CNS disorders**

| **Final  diagnosis** | **Sex;  Age (range)** | **Past medical**  **history** | **Clinical**  **picture** | **Ancillary**  **testing** | **Main reason for biopsy** | **Biopsy technique** | **Histological**  **diagnosis** | **Therapeutic  impact; response** |
| --- | --- | --- | --- | --- | --- | --- | --- | --- |
| 1. IgG4-RD-1 | M;  70-75 | Pancreatitis, cholangitis, organizing pneumonia | Subacute cognitive decline, gait disorder | Serum: IgG4↑. MRI: pachymeningeal enhan-cement. CSF: pleocytosis.  PET-CT*: normal | Unclear etiology, suspicion inflammatory CNS disorder | Open | Granulomatous inflammation, vasculitis.  IgG4/IgG ratio ↑ | IVMP + oral prednisolone, azathioprine; moderate repons |
| 2. IgG4-RD-2 | M;  35-40 | Schizo-phrenia | Headache, hemiparesis | Serum: IgG4↑. MRI: pachymeningeal enhan-cement, cerebral edema. CSF and CT*: normal. | Unclear etiology, suspicion inflammatory CNS disorder | Open | Xanthogranulomat-ous inflammation  with Toutonlike giant cells, IgG4/IgG ratio ↑ | IVMP + oral prednisolone, rituximab; significant respons |
| 3. Neuro-sarcoidosis-1 | F;  50-55 | none | Headache | Serum: SIL-2R normal.  MRI: leptomeningeal enhan-cement. CSF: pleocytosis PET-CT*: cardiac enhancement. | Differentian-  Iation between neurosarcoidosis and CNS-TB | Open | Granulomatous inflammation, no necrosis, no microorganisms. | IVMP, oral prednisolone, infliximab, cyclophosphamide; complete respons |
| 4. Neuro-sarcoidosis-2 | F;  50-55 | Pulmonary and cutaneous sarcoidosis. ITX: prednisolone, ixekizumab | Encephalo-pathy | Serum: SIL-2R↑. MRI: supratentorial enhancing parenchymal lesions. CSF: normal. PET-CT*: NA. | Disease progression during ITX | Open | Granulomatous inflammation, no microorganisms. | IVMP, oral prednisolone, adalinumab; moderate respons |
| 5. Reumatoid meningitis | F;  60-65 | Rheumatoid arthritis.  ITX: adalinumab, metho-trexate | Headache, hemiparesis | MRI: leptomeningeal enhancement.  CSF: pleocytosis, OCB+.  PET-CT: normal. | Disease progression during ITX | Open | Encephalitis and vasculitis, necrotizing dural granulomas. No microorganisms. | IVMP oral prednisolone, ritixumab; no information on response |
| 6. CLIPPERS-1 | M;  55-60 | none | Dysarthria, pyramidal syndrome | MRI: pontine and subcortical perivascular enhancement. CSF: normal. | Disease progression during ITX, atypical MRI | Stereo-tactic | Glial tissue, T cells, reactive changes. No tumor cells. | Tocilizumab; moderate repons |
| 7. CLIPPERS-2 | M;  50-55 | none | Gait disorder | MRI: right cerebellar space occupying lesion, multiple enhancing foci.  CSF and PET-CT: normal. | Suspicion of brain tumor | Stereo-tactic | Encephalitis, B- and T-cells. No tumor cells. | IVMP, oral prednisolone + azathioprine;  Moderate repons |
| 8. Tume-feactive MS | M;  40-45 | MS | Hemiparesis | MRI: space occupying enhancing lesion (left frontal) . CSF: normal. | Exclusion brain tumor | Stereo-  tactic | Demyelinisation | IVMP, natalizumab;  Moderate respons |
| 9. PNID-1: vasculitis/  encephalitis | M;  70-75 | Type 2 diabetes mellitus, hypertension | Hemiparesis, encephalo-  pathy | MRI:bilateral enhnacing cortical T2 hyper-intensities and diffusion restriction. MRA: vascular wall enhancement (M1).  CSF: pleocytosis, OCB+ PET-CT: normal | Unclear etiology, suspicion inflammatory CNS disorder | Open | Encephalitis, perivascular infiltrates (cuffing), B-cells and T-cells | IVMP, cylcophosph-amide, rituximab; no respons |
| 10. PNID-2: vasculitis/  encephalitis | M;  70-75 | None | Subacute cognitive decline | MRI: bilateral T2 enhancing hyper-intensities (cortical and deep gray matter), diffusion restriction. MRA/DSA: NA.  CSF: pleocytosis, OCB+ PET-CT: normal | Unclear etiology, suspicion inflammatory CNS disorder | Open | Perivascular infiltrate, predominantly T-cells and macrophages. | IVMP, cylcophosph-amide, rituximab; significant respons |
| 11. PNID-3: brainstem- encephalitis | M;  50-55 | Type 2 diabetes mellitus, hypertension | Subacute cognitive decline, polydipsia and polyuria | MRI: enhancing brainstem lesion, noduli pituitary stalk.  CSF and CT: normal. | Unclear etiology,  suspicion inflammatory CNS disorder | Stereo-  tactic | Encephalitis , predominantly T cells | IVMP, cyclophosp-  amide;  moderate respons |

CLIPPERS=chronic lymphocytic inflammation with pontine perivascular enhancement. CNS-TB=central nervous system tuberculosis DMT=disease modifying therapy. IgG4-RD=IgG4 related disease. ITX=immunotherapy. IVMP=intravenous methylprednisolone.MRA=magnetic resoncance angiography. MS=multiple sclerosis. NA=not applicable. OCB=oligoclonal bands. PNID=probable neuroinflammatory disorder. SIL-2R=soluble IL2 receptor.

**eTable 8:** **Overview of grade 1B and 2 complications**

| **Complication grade; no. (%) of all brain biopsies** | **Description** | **No. (%) within subcategory** | **Treatment** |
| --- | --- | --- | --- |
| 1B: Symptomatic, no treatment required (13/107; 12%) | Delirium  Transient focal deficit  Intracranial hemorrhage  Cerebral edema | 5/13 (38) 5/13 (38)  2/13 (15) 1/13 (8) | N.A |
| 2: Symptomatic, treatment required (6/107; 6%) | Wound infection  Seizures  Intracranial hemorrhage Cerebral edema | 2/6 (33) 2/6 (33) 1/6 (17)  1/6 (17) | Antibiotics  Adjustment of antiseizure medication Reversal of anticoagulation  Corticosteroids |

N.A.=not applicable.

**eTable 9: Description of patients with grade 4 complication (postoperative death within 30 days)**

| **Patient no. /  final diagnosis** | **Sex;  age (range)** | **Predisposing factors** | **mRS**  **(preopera-tive)** | **Biopsy  type** | **Duration**  **until complication (days)** | **Description** |
| --- | --- | --- | --- | --- | --- | --- |
| 1. Supratentorial lesion of unknown origin | F;  60-65 | ICU admission, thrombocyte-penia, sepsis | 5 | Stereotactic | 5 | Massive intracranial hemorrhage, poor prognosis;  Clearly biopsy-related |
| 2. Cerebral toxoplasmosis | F;  45-50 | Immunocompr-  omised status | 4 | Stereotactic | 29 | Sudden death with unknown cause;  Relation with biopsy unlikely |
| 3. Pontine glioma | M;  75-80 | Targeted brainstem lesion | 3 | Stereotactic | 1 | Sudden death with unknown cause;  Relation to biopsy unknown |

ICU=intensive care unit. mRS=modified rankin scale.

|  | **Grade ≤1b  complication N=98** | **Grade≥2**  **complication**  **N=9** | **P-value** |
| --- | --- | --- | --- |
| Female gender no. (%) | 43 (44) | 4 (44) | 0.62 |
| Age at biopsy in years (median; IQR; range) | 58; 51-70; 19-79 | 60; 46-74; 39-76 | 0.47 |
| Thrombocyte count prior to biopsy  (median x10^9/L; IQR; range) | 259; 212-321; 60-749 | 183; 125-269; 78-281 | 0.51 |
| Antiplatelet therapy prior to biopsy  no. (%) | 20 (20) | 2 (22) | 0.59 |
| Anticoagulant therapy prior to biopsy no. (%) | 15 (15) | 2 (22) | 0.59 |
| Modified Rankin Scale (mRS; median; IQR; range) | 3; 2-4; 1-5 | 4; 3-5; 1-5 | **0.048** |
| Biopsy technique no. (%)  stereotactic biopsy  open biopsy  Repeat biopsy no. (%) | 51 (52)  47 (48)  13 (13) | 6 (67) 3 (33)  0 (0) | 0.40 0.40  0.24 |
| Target of brain biopsy* no. (%)  cortical  cortico-meningeal  subcortical  deep gray matter  brainstem or cerebellum  enhancement | 47 (48) 15/47 (32) 38 (39) 5 (5)  8 (8)  77 (79) | 3 (33) 2/3 (67)  4 (44)  0 (0) 2 (22) 7 (78) | 0.40 0.22 0.74 0.64 0.20  0.62 |
| Duration between biopsy and complication in days (median; IQR; range) | 1; 0-2; 0-9 | 2; 1-10; 0-29 | 0.21 |

**eTable 10: Comparison of patients with no or minor (grade ≤1b) vs. major (grade ≥2) complications**

IQR=interquartile range.

|  | **No symptomatic intracranial hemorrhage N=103** | **Symptomatic intracranial hemorrhage  N=4** | **P-value** |
| --- | --- | --- | --- |
| Female gender no. (%) | 46 (46) | 1 (25) | 0.41 |
| Age at biopsy in years (median; IQR; range) | 58; 50-69; 19-79 | 66; 58-75; 57-76 | 0.60 |
| Thrombocyte count prior to biopsy  (median x10^9/L; IQR; range) | 257; 213-318; 60-749 | 153; 89-205; 78-212 | 0.13 |
| Antiplatelet therapy prior to biopsy  no. (%) | 22 (21) | 0 (0) | 0.39 |
| Anticoagulant therapy prior to biopsy no. (%) | 15 (15) | 2 (50) | 0.12 |
| Modified Rankin Scale (mRS; median; IQR; range) | 3; 3-4; 1-5 | 3; 2-4; 1-5 | 0.80 |
| Biopsy technique no. (%)  stereotactic biopsy  open biopsy  Repeat biopsy no. (%) | 55 (53) 48 (47) 13 (13) | 2 (50) 2 (50) 0 (0) | 0.64 0.64  0.49 |
| Target of brain biopsy* no. (%)  cortical  cortico-meningeal  subcortical  deep gray matter  brainstem or cerebellum  enhancement | 48 (47) 15/48 (32) 40 (39) 5 (5) 10 (10) 80 (88) | 2 (50) 2/2 (100)  2 (50)  0 (0) 0 (0) 4 (100) | 0.89 0.11 0.65 0.82 0.67 0.29 |

**eTable 11: Comparison of patients with no symptomatic intracranial hemorrhage vs. symptomatic intracranial
hemorrhage**

IQR=interquartile range.
